# Synergistic Antibacterial Effect and Mechanism of Allicin and an Enterobacter cloacae Bacteriophage

**DOI:** 10.1128/spectrum.03155-22

**Published:** 2022-12-06

**Authors:** Zhi Tao, Di Geng, Jiayue Tao, Jing Wang, Siqi Liu, Qiaoxia Wang, Feng Xu, Shengyuan Xiao, Rufeng Wang

**Affiliations:** a School of Life Sciences, Beijing University of Chinese Medicine, Beijing, China; b School of Pharmaceutical Sciences, Peking University Health Science Center, Beijing, China; c Engineering Research Center of Edible and Medicinal Fungi, Ministry of Education, Jilin Agricultural University, Changchun, China; Yangzhou University

**Keywords:** *Enterobacter cloacae*, allicin, bacteriophage, synergy, antibacterial mechanism

## Abstract

Enterobacter cloacae is a troublesome pathogen causing refractory infections of the lower respiratory tract, urethra and abdominal cavity, endocarditis, osteomyelitis, and neonatal septicemia. It is prone to developing resistance to ordinary antibiotics and has brought a serious problem to clinical treatment. An artful synergistic combination of an antibacterial natural product allicin and a newly isolated bacteriophage, named BD523, was constructed herein. This combination significantly lowered effective dosage of allicin and effectually overcame bacterial drug-resistance. We experimentally evidenced that allicin interacts with bacterial DNA in the groove region by inserting itself into the DNA double helix and, subsequently, disrupts the bacterial DNA by cleaving phosphate diester bonds of deoxynucleotides. Further, BD523 destroys the cell wall and membrane of bacteria by synthesizing lyase proteins, including holin and endolysins. Thus, the synergistic effect of the combination benefits from complementary targeting mechanisms of allicin and BD523. They cooperatively act on bacterial DNA, cell wall, and membrane to improve antibacterial efficiency and avoid drug-resistance.

**IMPORTANCE** Bacterial drug-resistance is a serious problem afflicting pharmacologists all over the world. Many strategies have been developed and practiced to overcome it, but almost no one is satisfactory due to the continual change of bacteria. Combinations of antibiotics and bacteriophages are promising because of the cooperation of 2 bacterial killers with distinct mechanisms. The combination of allicin and an Enterobacter cloacae bacteriophage reported herein can significantly improve the effect of allicin against E. cloacae. Its synergistic effect was even superior to the combination of bacteriophage and neomycin, of which the MIC was significantly lower than allicin. It was ascribed to the complementary antibacterial and the possible resistance-proof mechanism of bacteriophage and allicin. This study provided a pragmatic way to conquer the cunning bacterium, and may offer reference for research and development of new bacterial killers.

## INTRODUCTION

Enterobacter cloacae is a troublesome pathogen causing refractory infections of the lower respiratory tract, urethra and abdominal cavity, endocarditis, osteomyelitis, and neonatal septicemia (1). It is also a member of the six super-resistant bacteria ESKAPE (Enterococcus faecium, Staphylococcus aureus, Klebsiella pneumoniae, Acinetobacter baumannii, Pseudomonas aeruginosa and Enterobacter spp.) group resistant to more than 90 antibiotics such as penicillin, cefoxitin, amphotericin, and tetracycline. The bacterium resists antibiotics through regulating expression of proteins, including β-lactamase, membrane transporter, and efflux pump (2). For such refractory microbe, an effective solution is to use broad-spectrum antibiotics or new bacterial killers with novel antimicrobial mechanisms, such as natural products. Allicin, i.e., diallyl thiosulfinate ester, is a natural organic sulfur compound from garlic bulbs (3). It is effective against both Gram-positive and -negative bacteria including drug-resistant strains, e.g., Burkholderia cepacia, P. aeruginosa, Escherichia coli, S. aureus, and Helicobacter pylori (4–9). It has been used to cure lung infections and heal gastric ulcers caused by H. pylori (6, 9). Bacteria hardly develop resistance to allicin due to its disulfide bond formed with free sulfhydryl group, which changes protein spatial structure and makes enzymes inactive (10–12). Allicin also interacts with bacterial cell membranes and impairs their permeability and integrity (13). It was found that allicin and its analogues inhibit DNA gyrase of E. coli (14), but its immediate effect on bacterial DNA is unclear. Another solution for refractory microbes is using bacteriophage, which is mechanistically different from antibiotics. Bacteriophage specifically recognizes and eliminates host bacterium and thereby its use in overcoming bacterial infections is highly expected (15, 16). Unfortunately, bacteria also develop resistance to bacteriophages through, e.g., binding site mutation, adsorption inhibition, DNA penetration block, induction of bacteriophage DNA degradation, etc. (17–19).

Combinations of antibiotics are usually employed to deal with antibiotic-resistant bacteria. Nevertheless, because of similar shortcomings, they may eventually incur the same outcome as individual antibiotics alone (20). In contrast, concomitant use of antibiotics or natural products and bacteriophages seems to be a practical way (21). For example, a combination of rifampicin and a bacteriophage more effectively treated S. aureus infection than a combination of rifampicin and other antibiotics, such as azithromycin and vancomycin (22). Likewise, a combination of meropenem and a bacteriophage improved the survival rate of Galleria mellonella infected with B. cepacia at least 36 times as against the antibiotic and bacteriophage used alone (23). The combination of antibiotics or natural products and bacteriophages is promising due to the cooperation of bacterial killers with distinct antibacterial mechanisms; however, to what degree this combination improves the antibacterial efficiency and how these distinct mechanisms mutually benefit need to be elucidated (24, 25). Therefore, we used allicin and a newly isolated bacteriophage, BD523, as an example to investigate their synergistic effect and mechanism against E. cloacae.

## RESULTS

### Antibacterial effect and mechanism of allicin.

**FIG 1:**
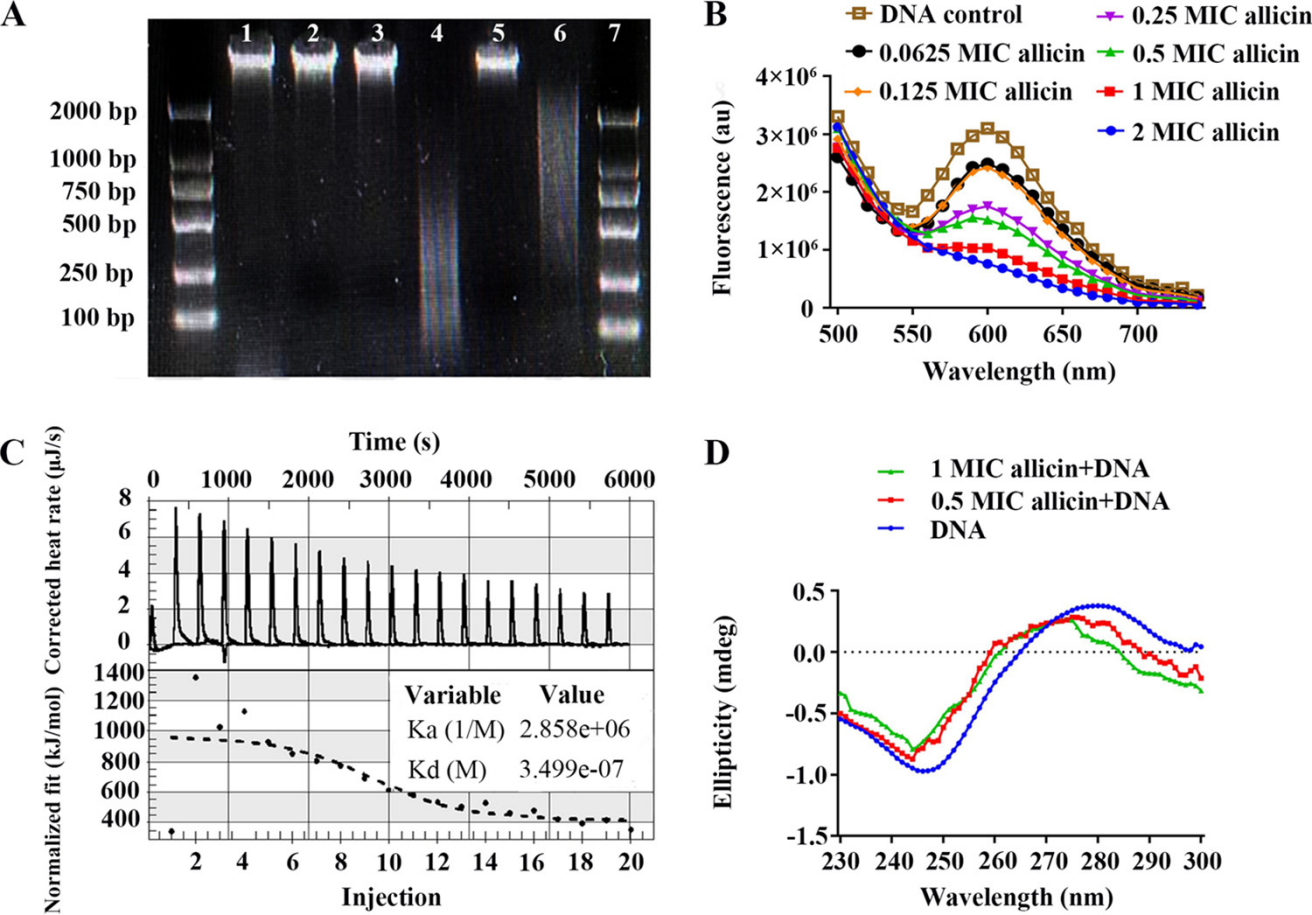
Mechanism of DNA degradation by allicin. (A) Agarose gel electrophoresis diagram of DNA degradation by allicin. Lanes: 1, Marker; 2, Blank control (a mixture of equal volumes of DNA and pure water); 3, BD523; 4, Neomycin; 5, Allicin; 6, Neomycin + BD523; 7, Allicin + BD523; 8, Marker. DNA trailing is only observed in lanes 5 and 7. (B) Allicin decreases fluorescence intensity of DNA-GelRed complex at 600 nm in a concentration dependent manner. (C) Isothermal titration calorimetry of interaction between allicin and DNA displays a positive binding peak and its height decreases gradually along with dilution of DNA. (D) Positive peak at 276 nm and negative peak at 245 nm in circular dichroism (CD) evidence right helix conformation and base accumulation of DNA, respectively. Height of the peaks decreases concentration-dependently with addition of allicin.

The MIC of allicin against E. cloacae, based on bacteriostatic zone, was 125 μg/mL, while that of a positive control neomycin was only 31.25 μg/mL (Table S1). Agarose gel electrophoresis showed that allicin reacted with DNA and resulted in DNA lag and degradation (Fig. 1A, lanes 5 and 7), whereas neomycin or bacteriophage BD523 alone or their combination did not give the reaction (Fig. 1A, lanes 3, 4 and 6). DNA-GelRed (a nucleic acid dye) fluorescence assay unveiled that allicin reduced fluorescence intensity of DNA-GelRed complex at 600 nm, shedding light on a competitive binding between allicin and GelRed to DNA (Fig. 1B). Isothermal titration calorimetry (ITC) evidenced binding of allicin to DNA by displaying a positive peak with △H < 0 and △S < 0. The peak height reduced gradually along with dilution of DNA, which is indicative of an exothermic process (Fig. 1C). Association constant (K_a_) and dissociation constant (K_d_) values were 10^6^ and 10^7^, respectively, which reflect a strong binding of allicin to DNA. A positive peak at 276 nm and a negative peak at 245 nm in circular dichroism spectrum (CD) decreased in an allicin concentration dependent manner (Fig. 1D), indicating that allicin interferes with right-handed helical conformation and base accumulation of bacterial DNA.

In interaction of allicin with bacterial DNA monitored by liquid chromatography tandem mass spectrometry (LC-MS/MS), deoxyadenosine (dA), and deoxyguanosine (dG) were found since their parent ions at *m/z* 252.1089 and 268.1038 (calculated for C_10_H_13_N_5_O_3_, 252.1091 and C_10_H_13_N_5_O_4_, 268.1040) were detected, respectively (Fig. S1A). In addition, fragmented product ions (*m/z* 136 and 152) for dA and dG were also observed in the secondary mass spectrum. Similarly, in a simulation test using allicin and deoxyribonucleotide triphosphates (dNTPs), which are basic construction units of DNA, all 4 deoxynucleosides were identified by detecting the respective parent ions and product ions, namely, at *m/z* 228.0974 (calculated for C_9_H_13_N_3_O_4_, 228.0979) and 112 for deoxycytidine (dC), *m/z* 268.1036 (calculated for C_10_H_13_N_5_O_4_, 268.1040) and 152 for dG, *m/z* 252.1087 (calculated for C_10_H_13_N_5_O_3_, 252.1091) and 136 for dA, and *m/z* 243.0964 (calculated for C_10_H_14_N_2_O_5_, 243.0975) and 127 for thymidine (T) (Fig. S1B). In contrast, none of such deoxynucleosides were detected in the control group without allicin added (Fig. S2).

Table S2 illustrates comparative antibacterial activities of allicin and its analogues (Fig. S3) evaluated by MIC in a potency descending order as ethylicin or methyl methanethiolsulfonate, allicin, diallyl disulfide, and 1,7-octadiene, providing a basis for structure-activity assessment.

### Isolation, identification, and biological characteristics of a bacteriophage.

**FIG 2:**
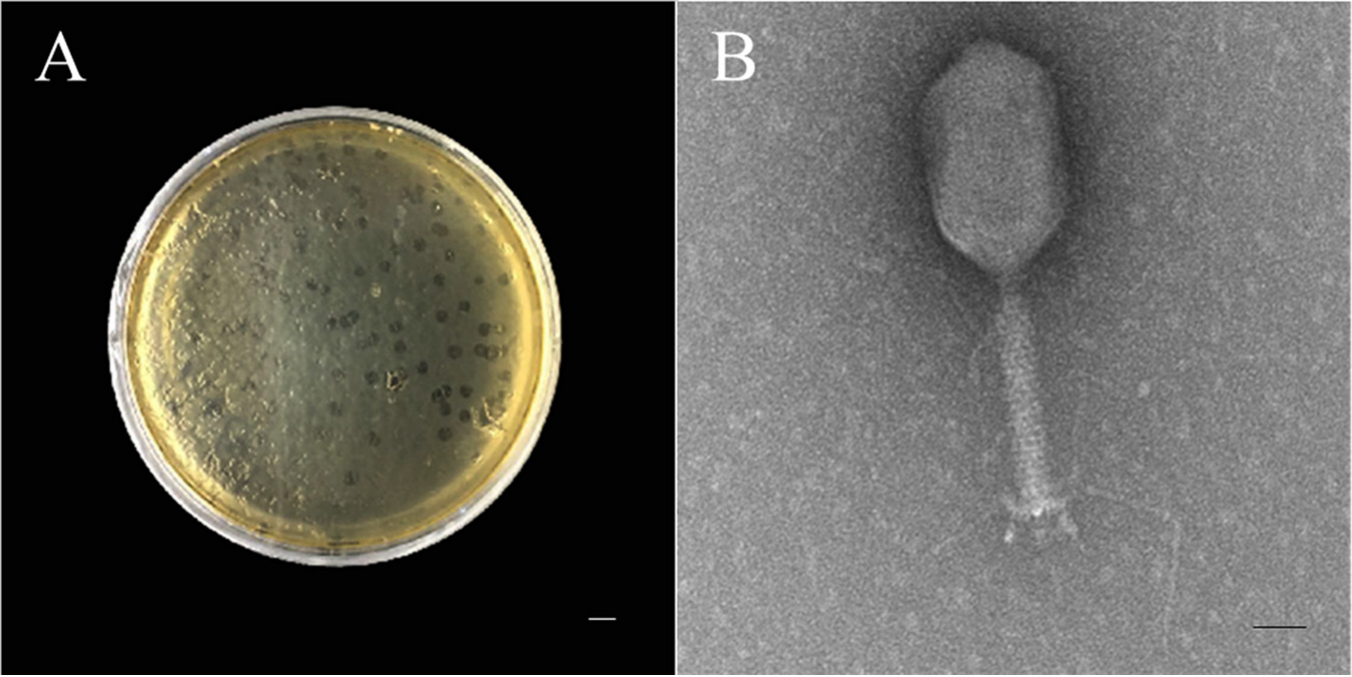
Macroscopic morphology of plaque and submicroscopic morphology of single bacteriophage of BD523. (A) Macrophotograph of the plaque. The bar is 1 cm long. (B) Transmission electron micrograph of a single bacteriophage with a regular polyhedral head and retractable long tail composed of baseplate and fibers connected by a neck strap. The bar is 50 nm long.

E. cloacae ATCC 13047 obtained from Shanghai Beinuo Biotechnology Co., Ltd. was used to screen the bacteriophage. The strain was shown to tolerate a lot of antibiotics, such as β-lactams, macrolides, aminoglycosides, tetracyclines, and quinolones. After an extensive screening, a bacteriophage against this strain was obtained and named BD523. It produced a plaque about 1 mm in diameter in solid medium (Fig. 2A). A single plaque was selected and added to the growth phase bacterial solution for enrichment culture to obtain a large number of bacteriophage. Transmission electron microscopy (TEM) electron micrographs displayed its regular polyhedral head of 99.7 ± 3.5 nm long and 72.7 ± 3.1 nm in diameter, and retractable long tail of about 99.3 ± 2.5 nm long with a baseplate and fibers (Fig. 2B). Considering the morphological features and the results of a phylogenetic tree showing high similarity of this bacteriophage to Escherichia phage IMM-002 (NC 048071.1) (Fig. S4B), the percentages of identity and coverage were 88.43% and 89%, respectively. BD523 was determined as the Caudoviricetes, Caudovirales, Autographiviridae, Studiervirinae, Kayfunavirus, Escherichia phage.

Based on plaque counting, bacteriophage titer was calculated as 1.2 × 10^9^ CFU/mL, and optimal multiplicity of infection (OMOI) was 0.1 (Table S3). According to a one-step growth curve (Fig. S5A), the latent period of BD523 was 0 to 30 min, and the rise period was 30 to 50 min. The amount of burst size was 1.58 × 10^3^ PFU/cell, and the optimum temperature range was between 35 and 40°C. Over 40°C, activity of BD523 attenuated inversely related to temperature (Fig. S5B). Optimal pH for BD523 was 7.0, and its titer decreased substantially beyond this value (Fig. S5C). After treatment with 10% chloroform, neither its titer nor its lysis ability obviously decreased (Fig. S5D).

*In vitro* antibacterial experiments of BD523 showed that OD600 values of experimental groups increased slowly at first, followed by a rapid decrease. Compared with the control group, the OD600 in different multiplicity of infection (MOI) groups reduced significantly within 80 min (Fig. S5E).

### Genomic analysis and lyase protein expression.

The genome of the bacteriophage consists of a double-stranded linear DNA of 39,686 bp with GC content of 52.7%. A genome annotation revealed that BD523 contained a total of 49 open reading frames (ORFs), including 7 for structural proteins, 19 for nonstructural proteins, and the rest for hypothetical or unknown proteins. Its genome (Fig. S4A) was composed of an invasion system (orange), replication system (green), assembly system (red), structural proteins (light blue), lysis system (purple), and hypothetical proteins (yellow). The lyase proteins, including holin and endolysins, were successfully expressed with recombinant plasmids constructed using 3 amplified lyase gene sequences (E, GP17.5, and GP18.5), and the plasmid pET22b vector (Fig. S6A and B). MICs of proteins E, GP17.5, and GP18.5 were determined to be 138 ± 11.4, 148 ± 9.5, and 107 ± 14.1 μg/mL, respectively.

### Synergistic effect and mechanism.

**FIG 3:**
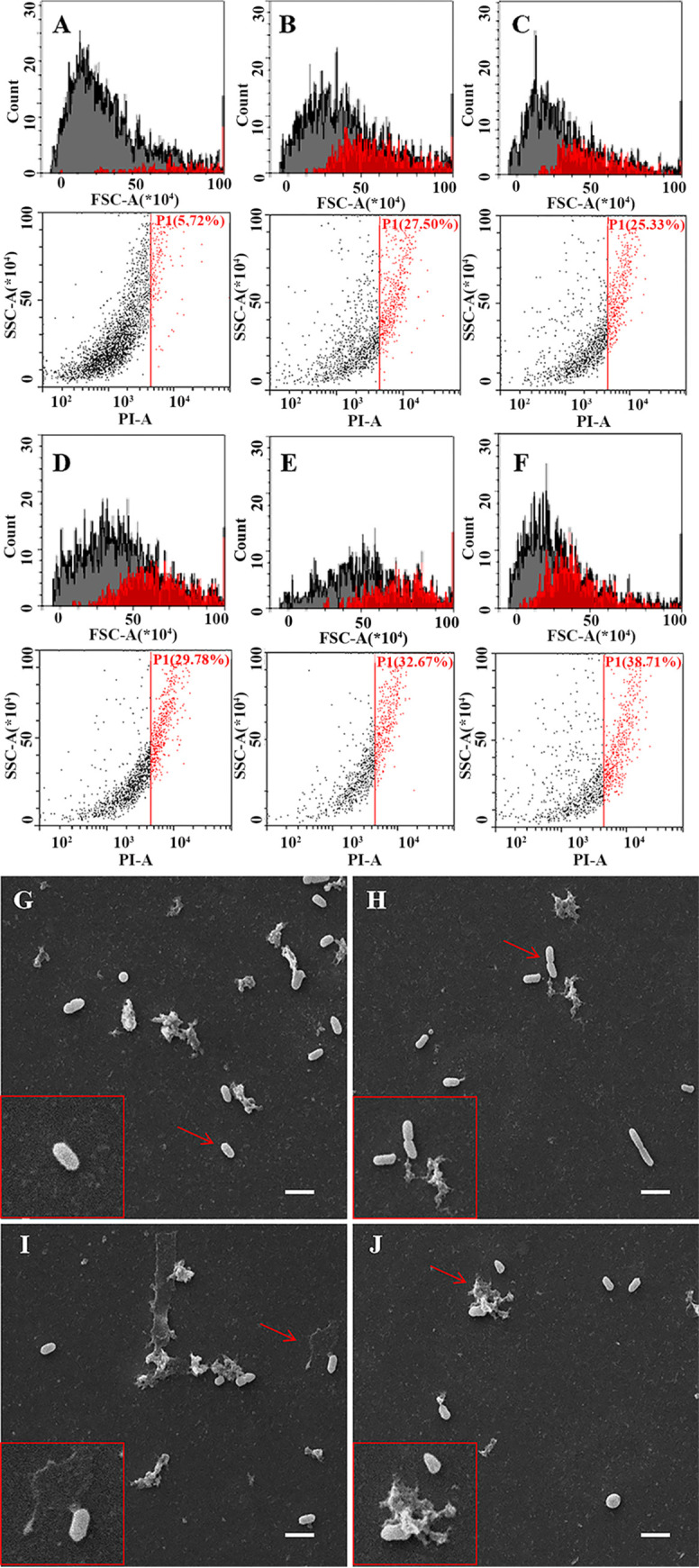
Flow cytometry histogram/scatter diagram and scanning electron micrograph of the treated E. cloacae. (A to F) represent flow cytometry histogram/scatter diagram, and (G to J) represent scanning electron micrograph of E. cloacae. (A) Treated with PBS, and PI staining rate is 5.72%. (B) Treated with BD523, and PI staining rate is 27.50%. (C) Treated with neomycin, and PI staining rate is 25.33%. (D) Treated with allicin, and PI staining rate is 29.76%. (E) Treated with neomycin + BD523, and PI staining rate is 32.67%. (F) Treated with allicin + BD523, and PI staining rate is the highest 38.71%. (G) Normal bacterium. (H) Bacterium with perforated cell wall and membrane and initially ejected cellular content. (I) Bacterium with lacerated cell wall and membrane and overflowed massive cellular content. (J) Collapsed bacterium. In panels (A to F), red represents dead bacteria and gray represents surviving bacteria, PI means propidium iodide, and PBS means phosphate-buffered saline. Scanning electron micrograph of E. cloacae treated with allicin and BD523. In panels (G to J), bars are 2 μm long.

A concomitant antibacterial test for allicin and BD523 proved that BD523 reduced effective concentration of allicin from 1 to 0.01 MIC (Fig. S7A). In the BD523 group, turbidifaction began to appear after 4 h, and the intragroup difference reached a maximum at 8 h (Fig. S7A, the red line). Antibacterial activity of allicin + BD523, ciprofloxacin + BD523, and polymyxin + BD523, especially the first 2 combinations, were stronger than that of other combinations including penicillin + BD523, neomycin + BD523, sulfadiazine + BD523, and metronidazole + BD523 (Fig. S7B). In addition, allicin + BD523 had a stronger and longer antibacterial effect than BD523 alone (Fig. S7C). Leakage of proteins and nucleic acids in all groups was positively correlated with time, and that in the allicin + BD523 group was higher than that in the allicin alone group (*P < *0.01) and neomycin + BD523 group (*P < *0.05). Nevertheless, no significant difference was observed between the neomycin + BD523 group and the neomycin alone group (Fig. S8A and B). Hydrophobicity of the bacterial wall and membrane was decreased time-dependently by BD523, allicin, and allicin + BD523. Within 1 to 5 h, the adsorption rate in allicin + BD group decreased most significantly, and was negatively correlated with time (*P < *0.05) (Fig. S8C). Membrane injury rate detected by flow cytometry was 27.5, 29.78, and 25.33% in BD523, allicin, and neomycin groups, respectively (Fig. 3B to D), which was significantly higher than that in the model group (Fig. 3A) (*P < *0.05). Moreover, this rate in allicin + BD523 group was as high as 38.71% (Fig. 3F). Scanning electron microscopy (SEM) electron micrographs intuitively exhibited the injury process of allicin + BD523 on the bacterial cell wall and membrane from normal (Fig. 3G) to perforated (Fig. 3H), lacerated (Fig. 3I), and, finally, total collapsed (Fig. 3J).

**FIG 4:**
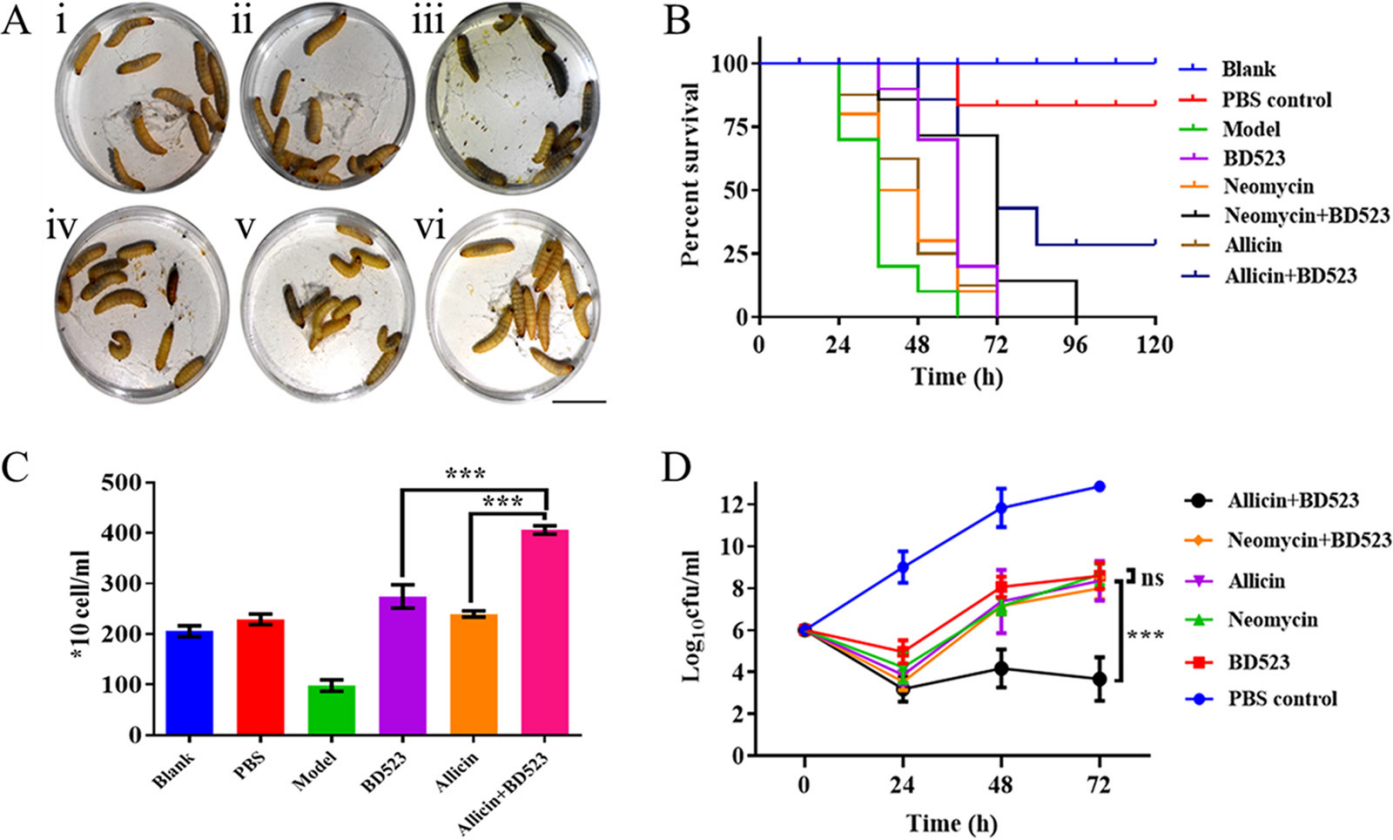
Appearance, survival rate, bacterial load and immune cell count of G. mellonella treated with antibacterial substances and BD523 subsequent to E. cloacae infection. (A) Appearance of larvae in each group, i, Blank control; ii, PBS control; iii, Model (given an inoculum of just E. cloacae); iv, Allicin; v, BD523; vi, Allicin + BD523. The larva is considered dead in the case it is full of black spots and does not respond to external stimuli. (B) Survival rates of larvae in different groups within 120 h, *n *= 10. The value at 48 h after treatment is selected for each group, which shows significant difference in efficacy. (C) Blood lymphocytes of larvae in each group counted at 12 h after treatment. (D) Bacteria load of larvae in each group in 72 h. Allicin + BD523 shows the strongest inhibition of bacterial proliferation. The experiments were carried out in triplicate and results are shown as mean ± SD. Statistical analysis was performed by One-way ANOVA. *** means *P < *0.001 compared with the group connected by line, and ns means statistically insignificant difference. Bar is 2 cm long. PBS means phosphate-buffered saline.

In *in vivo* antibacterial experiments using G. mellonella, the larvae in the blank control and PBS groups showed no obvious appearance change, whereas those infected with E. cloacae showed black spots and was insensitive to external stimuli within 120 h (Fig. 4A). The survival rate of larvae at 48 h in the model group decreased to 10%, whereas it increased significantly (*P < *0.05) in the treatment groups, especially in the allicin + BD523 group as it was up to 80% (Fig. 4B). Blood lymphocyte counting at 12 h uncovered that allicin + BD523 stimulated and produced more immune cells than allicin or BD523 alone (Fig. 4C). The results of lymphocyte morphology also showed that the combined group could effectively reduce the number of bacteria and enhance immunity (Fig. S9). In a bacterial load test, allicin + BD523 effectively suppressed bacterial proliferation within 72 h. As a comparison, the bacterial load in the other treatment groups recovered at 48 or 72 h (Fig. 4D).

**FIG 5:**
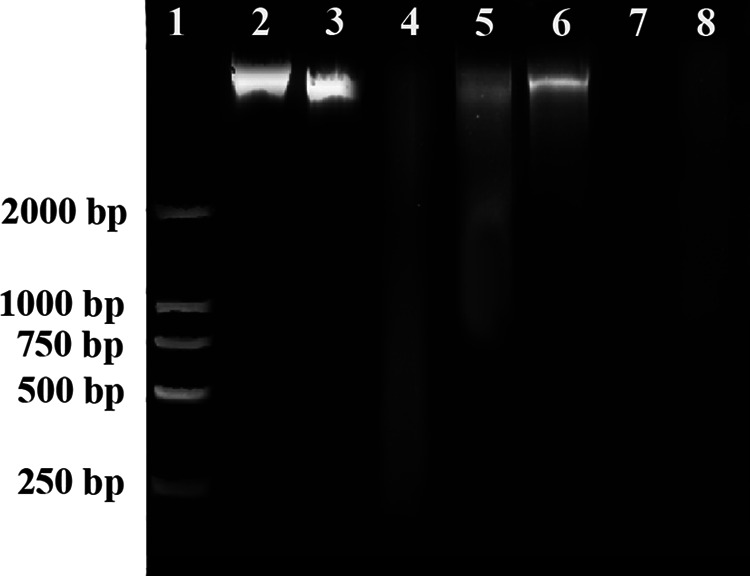
Agarose gel electrophoretic diagram of DNA degradation by BD523 and allicin. Lane: 1, Marker; 2, Blank; 3, BD523; 4, 1 MIC allicin; 5, 0.1 MIC allicin; 6, 0.01 MIC allicin; 7, 0.1 MIC allicin + BD523; 8, 0.01 MIC allicin + BD523.

An agarose gel electrophoresis test for bacterial DNA extracted after administration (Fig. 5) showed that allicin under MIC or BD523 alone did not degrade or weakly degrade bacterial DNA (Fig. 5, lanes 3, 5, and 6). In contrast, the combination of allicin and BD523 significantly enhanced the degradation of DNA (Fig. 5, lanes 7 and 8).

## DISCUSSION

Concomitant use of antibiotics or natural products and bacteriophages is a pragmatic way to overcome antimicrobial drug-resistance. Elucidation of their synergistic mechanism is the key to direct clinical use and new agent development. Mutual complementation of their mechanisms may be preferentially considered for optimizing the combination of antibiotics and bacteriophages.

We noticed herein that the effect of allicin against E. cloacae was moderate, but it was far weaker than neomycin. Allicin acts on bacteria through binding to DNA that has been proven by our ITC test and DNA-GelRed fluorescence assay. Strong binding of allicin to DNA reflected by large K_a_ and K_d_ values from ITC indicates that allicin interacts with DNA in the groove region and inserts itself into the DNA double helix in a non-classical way (26). GelRed embeds itself into base pairs of DNA to produce high fluorescence intensity, while allicin reduces the fluorescence intensity of DNA-GelRed complex through competing with GelRed. This further supports that allicin binds to DNA in the groove region. Change of CD characteristic peaks shows that allicin interferes with DNA right-handed helical conformation and reduces base accumulation. Trailing of electrophoretic bands demonstrates that degradation of DNA is one of the antibacterial mechanisms of allicin. It is confirmed by deoxynucleosides detected by LC-MS/MS in the interaction between allicin and bacterial DNA or dNTPs. Detection of deoxynucleosides also shows that allicin degrades DNA by breaking phosphate diester bonds.

Based on MICs of allicin and its analogues, we have established a structure-activity relationship and uncovered the antibacterial pharmacophoric group of allicin. Firstly, the terminal double bond of allicin is almost unrelated to antibacterial activity because 2 analogues with this moiety e.g., 1,7-octadiene and diallyl disulfide, are far less potent than allicin while another 2 analogues without this moiety, e.g., ethylicin and methyl methanethiolsulfonate, are more potent than allicin. Secondly, antibacterial potency of compounds with moieties of thiosulfone (
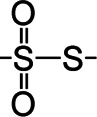
), thiosulfoxide (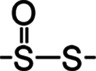), disulfide (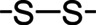), and ethylene (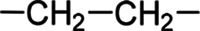) decreases in order. Among them, analogues with disulfide moiety are more potent than those with ethylene moiety. Thirdly, analogues containing thiosulfone moiety or thiosulfoxide moiety seem more potent than those containing disulfide moiety. Thus, the oxygen atom of moieties of thiosulfone and thiosulfoxide is unusually important for antibacterial activity. Both thiosulfone and thiosulfoxide are stronger nucleophilic groups due to unshared pair electrons of the oxygen atom. Disulfide moiety is in favor of increasing electron density of the oxygen atom to intensify nucleophilic capacity of thiosulfone and thiosulfoxide. Therefore, we deem thiosulfoxide moiety to be the major antibacterial functional group of allicin.

With these in mind, we may deduce the reacting process of allicin with bacterial DNA, as below. With its lone pair electrons of the oxygen atom, thiosulfone moiety of allicin abstracts a hydrogen proton from H_2_O to form OH^−^. The latter attacks the electrophilic phosphorus atom of the dihydrogen phosphate bond of nucleotides through a S_N_2 like nucleophilic substitution, which results in opposite moiety leaving, and eventually entails phospho-oxy bond breaking.

We found that BD523 has a strong lysis effect and good stability by detecting its biological characteristics such as titer, OMOI, one-step growth curve, optimal temperature, optimal pH, and chloroform tolerance. Host bacterium E. cloacae was killed within 80 min by BD523 even at non-OMOI. The antibacterial effect of BD523 is ascribed to lyase proteins, which has been verified by our antibacterial test. Holin encoded by GP17.5 is responsible for forming pores on the host cell membrane, and endolysins encoded by E and GP18.5 overflow outside membrane through the holes and, subsequently, cleave the cell wall by degrading peptidoglycans. However, we also observed a high back-turbidity rate reflected by larger SD values after 8 h (Fig. S7A), since E. cloacae is inclined to mutate into a tolerant strain in response to stress of BD523. Further, we isolated the resistant strain from the turbid lysate, which was not lysed by BD523. Similarly, the stress of bacteriophages also facilitates development of antibiotic-resistant bacteria. It was reported that the bacteriophages that preferentially target antibiotic-sensitive bacteria may promote antibiotic-resistant subpopulations through a phenomenon similar to “competitive release” observed in antibiotic combinations (27).

To understand the combined antibacterial effect of allicin and BD523, we employed both *in vitro* and *in vivo* antibacterial tests. The *in vitro* test showed that the combination significantly reduces effective concentration of allicin, increases leakage of proteins and nucleic acids, enhances injury of the membrane, attenuates hydrophobicity of the bacterial wall and membrane, as well as depresses back-turbidity. Besides the apparent effect of reduced effective concentration of allicin, the increased proteins and nucleic acids leakage and membrane injury accelerated the death of bacteria; where the attenuated bacterial wall and membrane hydrophobicity made bacteria less adhesive to decrease their infectivity, and the depressed back-turbidity demonstrated the possible resistance-proof effect. BD523 may help allicin enter host cells and degrade DNA efficiently. These results imply a synergistic antibacterial effect of the combination. The *in vivo* experiment using G. mellonella also provided similar results. The combination significantly improved survival rate of the larvae, and significantly prolonged their survival time (*P < *0.001) (Fig. 4B).

**FIG 6:**
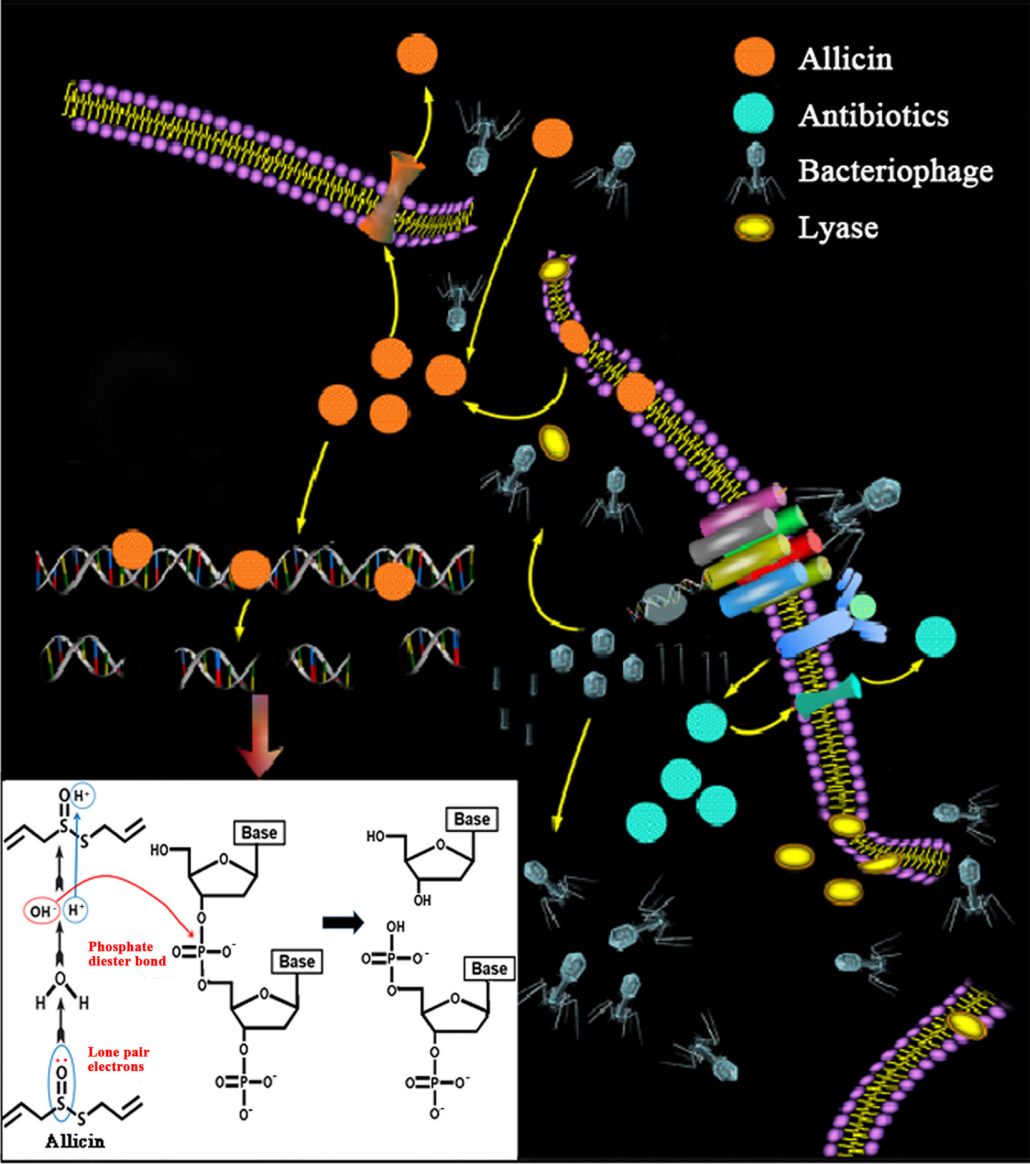
Overview of synergistic antibacterial mechanism of allicin and BD523. Allicin easily enters bacterium through holes or ruptures on bacterial cell wall and membrane produced by lyases of BD523 and interacts with bacterial DNA in groove by inserting itself into DNA double helix and subsequently disrupts bacterial DNA by cleaving phosphate diester bonds of deoxynucleotides. The diagram at the lower left shows the breaking mechanism of phosphate diester bond.

It is generally believed that antibiotics or natural products and bacteriophages double-target specific bacterial targets, reducing the probability of drug-resistance and bacterial variation. The combination also reduces dosage of antibiotics and severity of disease (20, 24). By comparing combined antibacterial effects of antibiotics that possess different mechanisms with BD523, we have noted that ciprofloxacin, which acts on DNA or polymyxin that acts on the cell membrane, has a synergistic effect with BD523 (Fig. S10). Based on this, we hypothesize that the synergistic effect of allicin and BD523 is probably related to the above specific mechanisms. The synergistic mechanism may be proposed as shown in Fig. 6. To start with, BD523 recognizes and binds to specific sites of the bacterial cell wall or membrane and then injects DNA into the host. After the DNA is replicated, the bacteriophage uses materials of the host to synthesize its own proteins and assemble them into progeny bacteriophages. Secondly, holin and endolysins produced by the progeny bacteriophages perforate and lacerate the host cell wall and membrane in the form of “internal lysis” (28). This action just helps allicin, which has difficulty crossing the cell membrane of the bacterium due to low polarity (ALogP = 1.5), enter the host cell and accelerates its contact with bacterial DNA. The bacteriophage may also inhibit host protein expression without damaging the DNA, as it facilitates the binding of allicin to the host DNA. Finally, the bacterium is totally killed by complementary attacks of both allicin and the bacteriophage.

To prove this conjecture, the degradation of DNA inside the bacteria after treatment with allicin or BD523 alone and their combination was studied. The results (Fig. 5) showed that the combination substantially enhanced the degradation of DNA in comparison with allicin or BD523 used alone. This confirmed the antibacterial synergetic effect and mechanism of allicin and BD523 as we proposed.

Interestingly, a positive drug, neomycin, did not exhibit such a potent synergistic effect with BD523 (Fig. S7C), although it showed a stronger activity (MIC 31.2 μg/mL) than allicin (MIC 125 μg/mL) when used alone (Table S1). The reason may be that neomycin, as one of the aminoglycoside antibiotics, interferes with protein synthesis through binding to bacterial ribosomes. This process may coincidentally hamper production of holin and endolysins, or hinder proliferation of the bacteriophage (29). Thus, the antibiotics or natural products not targeting proteins are preferentially recommended to pair with bacteriophages in order to maximize their complementary effects. This study provided a basis for the selection of potential compounds that synergistically combine with bacteriophages, and underlay the development of antibacterial agents.

In summary, the combination of allicin and BD523 has a synergistic effect due to their complementary targeting mechanisms. They cooperatively act on bacterial DNA, cell wall, and membrane so as to improve antibacterial efficiency and avoid drug-resistance.

## MATERIALS AND METHODS

### Determination of MIC.

Stock solutions of antibiotics were prepared by dissolving 1 mg each of allicin, neomycin, or vancomycin into 1 mL of phosphate-buffered saline (PBS), and serially added into bacterial solution (10^6^ CFU/mL) to give final concentrations of these antibiotics as 15.6, 31.2, 62.5, 125, and 250 μg/mL, respectively. Minimum concentration that completely suppressed bacterium growth after 8 h was regarded as MIC. The Kirby Bauer method was employed to evaluate the antibacterial effect of antibiotics. Sterilized 5 mm circular paper slips were dripped with the above serial solutions and then placed on a plate coated with bacterial suspension. After they were cultured in an incubator for 16 h, the diameter of the inhibition zone was measured (30).

### Observation on bacterial DNA injury.

Bacterial DNA was extracted using a DNA extraction kit manufactured by Beijing Biomed Gene Technology Co., Ltd. per its protocol. The extracted DNA at a concentration of 50 μg/mL was divided into 6 aliquots subsequent to purity qualification. To each aliquot, pure water, BD523, 1 MIC neomycin, 1 MIC allicin, 1 MIC neomycin + BD523, and 1 MIC allicin + BD523 were added in equal volume, respectively. After they were cultured under shaking at 37°C for 30 min, 10 μL of each aliquot was taken, and DNA migration was observed on 1% agarose gel electrophoresis.

### DNA binding mode.

The extracted DNA was diluted to 50 μg/mL, and each 5 μL along with 10 μL of 100 μg/mL GelRed were added to each well of the black hole plates. After mixing, 50 μL of allicin at different concentrations was added. In the control group, 50 μL of pure water instead of allicin was used. All samples were incubated at 37°C for 30 min away from light, and then fluorescence intensity was determined using a multifunctional microplate analyzer at an emission wavelength of 600 nm. An interaction curve of allicin with DNA was obtained by a titration thermal test. During the test, 300 μL of DNA was placed in a tank and 50 μL of allicin was loaded into a needle. After a delay of 900 s, titration started. The whole volume of about 20 drops in the needle was added to the tank, drop by drop, at intervals of 180 s under a stirring speed of 200 r/min. In the reference tank, the same volume of DNA solution was added. NanoAnalyze software (version 2.4.1) was used for data fitting analysis, and ITC Run software (version 3.4.1.0) was used for obtaining thermodynamic parameters and fitting images. Binding mode of allicin to DNA was explored using circular dichroism spectrometer. Allicin solutions of 0.5 and 1 MIC were incubated with 10 mM DNA at 37°C for 30 min, respectively. Pure water was used as control. The measurement wavelength was set at 230 to 300 nm with a bandwidth of 1 nm. The sampling interval of a single data point was 0.5 s, and data were exported by Chirascan software (version 4.0) (26).

### Elucidation of DNA injury mechanism.

**(i) Sample preparation.** About 10 μL of bacterial DNA (50 μg/mL) and 10 μL of 10 mM dNTPs were separately mixed with equal volume of 1 MIC allicin, and incubated overnight at 37°C. Three times the volume of methanol was added and a 0.22 μm microporous filter membrane was used to collect filtrate. Products were analyzed by liquid chromatography tandem mass spectrometry (LC-MS/MS) to determine cleaving position. DNA and dNTPs solution in pure water was used as control.

**(ii) LC-MS/MS analysis.** Each 3 μL of sample was injected into an Acquity UPLC BEH C18 column (100 mm × 2.1 mm, 1.7 μm) and analyzed at oven temperature of 35°C on a Thermo Q-Exactive high resolution mass spectrometer (Thermo Scientific) equipped with an electrospray ionization (ESI) interface source. Gradient elution was performed using 0.1% formic acid solution in pure water as mobile phase A and methanol as mobile phase B, at a flow rate of 0.3 mL/min with an elution program consisting of 10 to 90% B at 0 to 18 min, 90 to 10% B at 18 to 20 min, and constant 10% B at 20 to 25 min. Injection volume for each run was 3 μL. The ionization source was set to positive ion mode to obtain a total ion flow diagram of ESI-MS. The scanning range was 100 to 1000 *m/z*, and electrospray voltage and collision energy were set at 5500 and 20 V, respectively. Pyrolysis process was inferred through literature review, database retrieval, and analysis of pyrolysis rule by mass spectrometry. Relevant parameters of 4 deoxynucleosides are shown in table S4.

### Functional group identification.

The antibacterial functional group of allicin was identified by comparing the MICs of allicin and its structural analogues, including ethylicin, 1,7-octadiene, methyl methanethiolsulfonate, and diallyl disulfide determined with the Kirby Bauer method. All these compounds have similar linear skeletons but differ in functional groups. Thus, the active functional group of allicin was identified through inter-comparing the MICs of these compounds.

### Bacteriophage isolation.

Two grams of fresh rat feces were selected and placed in 10 mL of lysogeny broth (LB) medium. They were extruded and mixed, incubated at 37°C for 2 h, and centrifuged at 8000 r/min for 10 min. Then, the supernatant was filtered through a 0.22 μm filter membrane to obtain total bacteriophage solution. The solution was dropped into semi-solid medium containing host bacterium for inverted culture at 37°C for 12 h using double agar plates. In the case that potent bacteriophage of the host bacterium was present, plaque would be observed in solid culture medium. The plaque with good morphology was further purified and cultured three times. Purification was completed as long as the plaque with uniform morphology and consistent size was obtained. The plaque was picked and added to a sterile medium containing 7% dimethyl sulfoxide (DMSO). Then, it was mixed and frozen at −80°C (23, 31–33).

### Transmission electron microscopy.

The bacteriophage supernatant was collected in the same way as that described in the bacteriophage isolation section. An ultracentrifuge (Beckman Coulter, Inc.) was used for enrichment of BD523 at 30000 r/min for 2 h. The precipitate was mixed with 20 μL of ultrapure water. Then, 10 μL of bacteriophage suspension (10^9^ PFU/mL) was placed on a Formvar/carbon film coated grid and stayed for 10 min on a filter paper. A drop of 1% (wt/vol) uranyl acetate (pH 4.5) (Sigma-Aldrich [Shanghai] Trading Co., Ltd.) was dropped onto the grid surface for 90 s. After dying, the grid was absorbed by the filter paper, and then clamped on the filter paper to dry for 3 h. The grid was reloaded at 120 kV acceleration voltage onto a JEM-1400 Plus transmission electron microscope (JEOL USA Inc.) with image capture software (iTEM and KeenView Soft Imaging System).

### Genome sequencing and analysis.

The genome of bacteriophage was sequenced by Shanghai Tanpu Biotechnology Co., Ltd. using the Illumina NextSeq500 sequencing system manufactured by Illumina, Inc. The genomic sequence presented as double-stranded DNA of 39,686 bp with GC content of 52.7%, and was preliminarily determined by ORFs using RAST online database (https://rast.nmpdr.org/). All ORFs were verified using NCBI protein alignment tool BLASTp (www.ncbi.nlm.nih.gov/BLAST/). Whole genome circle mapping was completed using SnapGene (www.snapgene.com) (34, 35). To determine the phylogenetic relationship between BD523 and other families, top 10 genome sequences of NCBI BLAST were downloaded, and the phylogenetic tree was constructed using MEGA (version 7.0). Phylogenetic analysis was carried out using Clustal W and neighbor-joining methods (34, 35).

### Ultrastructure and cluster analysis.

A drop of bacteriophage solution was suspended on a copper grid. After being naturally precipitated for 10 min, it was fixed with 1% osmium acid and stained with 2% phosphotungstic acid. Then, it was naturally dried for 30 min in the dark. Finally, morphological characteristics such as head, tail, tail sheath, and base plate of the bacteriophage were observed by TEM.

### Biological characteristics of bacteriophages.

**(i) Bacteriophage titer assay.** Bacteriophage titer refers to the number of bacteriophages per milliliter of fluid. Plaque was picked with sterile spear and blown into medium to obtain stock solution of bacteriophage. Then, the stock solution was 10-fold serially diluted to 8 wells of a 96-well plate, and 10 μL of host bacterium solution was added to each well and fully mixed. This process was repeated 3 times. The mixture was cultured upside down using double agar plates at 37°C for 8 h, and then bacteriophage titer was obtained by counting plaque in the plate.

**(ii) OMOI determination.** OMOI means multiplicity of infection with the highest titer. MOI refers to ratio of bacteriophage to bacterium. Bacteriophage and host bacterium at a concentration of about 6 × 10^8^ CFU/mL were mixed in accordance with MOI of 1, 0.1, 0.01, 0.001, 0.0001, and 0.00001, and cultured for 1 h. Then, bacteriophage titer was determined.

**(iii) One-step growth curve plotting.** Host bacterium was mixed with BD523 at OMOI, and 100 μL was removed at 0, 10, 20, 30, 40, 50, 60, 70, 80, and 100 min (Fig. S5A). Bacteriophage titer of the supernatant was measured after centrifugation. The experiments were repeated three times in each group, and the average value was taken. Then, a one-step growth curve was drawn according to measured results. Based on the determined bacteriophage titer, the amount of bacteriophage burst was calculated using the following formula: amount of bacteriophage burst = bacteriophage titer at the end of burst/concentration of bacterium at the beginning of infection (36).

**(iv) Optimum temperature determination.** About 100 μL of bacteriophage solution was incubated in a water bath at 30, 40, 50, 60, 70, and 80°C, and sampled at 30 and 60 min, respectively. Then, bacteriophage titer was determined. The process was repeated three times and the average was taken.

**(v) Optimum pH determination.** About 100 μL of bacteriophage solution was mixed with LB medium at pH 3 to 12, respectively, and incubated in a water bath at 37°C for 1 h. Then, bacteriophage titer was determined. The process was repeated three times and the average was taken.

**(vi) Tolerance to chloroform test.** About 1 mL of bacteriophage solution was added into 9 mL of chloroform and the mixture was fully mixed. The solution was placed at room temperature for 30 min; then, upper solution was removed for titer determination. The process was repeated three times and the average was taken.

### Bacteriostatic test *in vitro*.

Bacteriophage solution was diluted into LB medium at different MOI, and 100 μL of host bacterium solution of 6 × 10^8^ CFU/mL was added. Then, OD600 value was measured at different time points within 80 min.

### Separation and purification of lyases.

**(i) Lyase recombinant plasmid construction.** Primers (Table S5) were designed and 3 segments of lyase gene sequences (E, GP17.5, and GP18.5) were amplified by PCR. The PCR protocol was as follows: denaturation at 94°C for 5 min followed by 25 cycles of 10 s at 94°C, 10 s at 55°C, 30 s at 72°C, and 5 min at 72°C. The amplified sequence and plasmid pET22b vector were subjected to double digestion (sites NdeI and XhoI), and were then connected to construct recombinant plasmids containing histidine label.

**(ii) Target protein expression.** Recombinant plasmids pET22b-E, pET22b-GP17.5, and pET22b-GP18.5 were transferred into competent E. coli BL21(DE3) by electroporation, respectively, and then expressed in BL21(DE3) cells to obtain recombinant proteins. At logarithmic growth phase, 1 mM isopropyl-β-d-thiogalactoside (IPTG) was added to induce expression overnight at 25°C, and then the bacterium was collected by centrifugation. The collected bacterium was resuspended and broken in an ice bath with a cell fragmentation device. Total supernatant proteins were collected by centrifugation, and target proteins were purified on the basis of specificity of NI-IDA magnetic beads with histidine label. Then, 20 μL of each purified target protein was used in bicinchoninic acid (BCA) protein assay kit (Phygene Biotechnology Co., Ltd.) for concentration determination, and calibration curve finally established was Y = 0.1575X + 0.0193, *r *= 0.9955 (X means protein concentration and Y means absorbance value). Purified lyases were successively diluted 5-fold with bacterial solution at logarithmic growth stage (10^6^ CFU/mL). Minimum concentration that completely inhibited bacterial growth after 8 h was considered to be MIC.

### Nucleic acids and proteins leakage detection.

Bacterial solution at logarithmic growth stage (10^8^ CFU/mL) was divided into 6 aliquots of 1 mL. The aliquots were centrifuged, suspended, and diluted to 10^6^ CFU/mL. Then, they were added with sterile water, 10 μL of BD523 with a titer of 10^9^ PFU/mL, 1 MIC neomycin, 1 MIC allicin, 1 MIC neomycin + BD523, and 1 MIC allicin + BD523, respectively, to allow final reaction system to be 0.5 mL. Next, the aliquots were cultured under shaking at 37°C, and sampled at 2 and 4 h, respectively. After that, absorbance of supernatant was measured at 260/280 nm by ultra-micro Uv-Vis spectrophotometer (Thermo Fisher Scientific [China] Co., Ltd.), and contents of nucleic acids and proteins leaked were calculated.

### Bacterial surface hydrophobicity determination.

Bacterial suspension of 10^8^ CFU/mL was prepared by mixing bacterial precipitation with 0.1 mol/L KNO_3_ solution. Each 100 μL of bacterial suspension was mixed with 100 μL of BD523, 1 MIC allicin, 1 MIC neomycin, 1 MIC allicin + BD523, and 1 MIC neomycin + BD523, respectively, as experimental samples, and another 100 μL of bacterial suspension was mixed with the same volume of pure water as control. After they were culture at 37°C for 12 h, OD405 value was measured and denoted as A0. Then, 1 mL of bacterial suspension was removed and 200 μL of cetane was added to it. The solution was vortexed vigorously for 2 min. After suspension was completely stratified, the OD405 value of aqueous phase was determined and denoted as A1. Surface hydrophobicity of bacterial cells was expressed by the following formula: adsorption rate% = 100 × (1 − A1/A0).

### Wall and membrane integrity observation.

Logarithmic bacterial precipitate was suspended and diluted to 10^6^ CFU/mL with PBS; then, it was added with sterile water, BD523, 1 MIC neomycin, 1 MIC allicin, 1 MIC neomycin + BD523, and 1 MIC allicin + BD523, respectively. After mixing, the bacterium was incubated at 37°C for 2 h. Then, it was stained with 50 μg/mL propidium iodide (PI). The impaired bacterium stained with PI was analyzed by flow cytometer (Beckman Coulter, Inc.). A drop of bacterial solution of allicin + BD523 group was added to a sterile cover glass for 30 min so that the bacterium could adsorb onto the glass as much as possible. Electron microscope fixative was added to fix the bacterium for 10 min, and was then sucked dry. After ethanol gradient dehydration and vacuum drying, platinum staining was sprayed. Finally, the prepared specimen was observed by scanning electron microscope FEI INSPECT S50 with image capturing software ThruSight and MAPS.

### Synergistic antibacterial test.

**(i) *In vitro.*** To investigate synergistic antibacterial effect of combination, 100 μL of bacterial solution at logarithmic stage was dropped into 10 mL of LB medium and mixed. The resulting solution was divided into 6 aliquots and used for preparing samples of model, bacteriophage, 0.5 MIC allicin, 0.5 MIC neomycin, 0.5 MIC allicin + bacteriophage, and 0.5 MIC neomycin + bacteriophage. A blank control using PBS was also set. Each group contained 3 duplicate samples. The samples were cultured under shaking at 37°C for 24 h, and then the OD600 value was measured at 0, 4, 8, 16, and 24 h.

To investigate influence of bacteriophage on antibacterial effect of allicin, a PBS blank group, model group, bacteriophage group, 1 MIC allicin group, 1 MIC allicin + bacteriophage group, 0.1 MIC allicin + bacteriophage group, 0.01 MIC allicin + bacteriophage group, and 0.001 MIC allicin + bacteriophage group were set. Each group contained 3 duplicate samples. The samples were cultured under shaking at 37°C for 12 h, and then the OD600 value was measured at each time point. In order to confirm the synergistic effect and mechanism of allicin and bacteriophage on DNA degradation, the above groups were incubated at 37°C for 24 h, and then bacterial DNA was extracted from each group for agarose gel electrophoresis.

**(ii) *In vivo.*** G. mellonellas fed in a dark environment at 18°C was used for synergistic antibacterial test *in vivo*. The larvae, which were more than 1 cm long and weighing 250 to 300 mg with uniform color and good vitality and without black or gray spots on the body surface, were selected as qualified experimental subjects. Control groups were divided into a blank control and PBS control. All selected larvae, except those in the control groups, were infected with 10 μL of bacterial solution of E. cloacae at a concentration of 10^6^ CFU/mL and divided into 6 groups, including model, BD523, 1 MIC vancomycin, 1 MIC allicin, 1 MIC vancomycin + BD523, and 1 MIC allicin + BD523. Each group contained 10 larvae. The bacterium was injected into the larvae through the left, lower hind foot using a microsampler. After injection, the larvae were placed in a 26°C incubator. In the case it did not respond to touch stimulation, it would be considered dead (37). The survival of each group was recorded within 120 h and a survival curve was drawn accordingly.

Bacterial load at different time periods was measured and blood lymphocytes were counted at 12 h. The larvae were placed in a centrifuge tube on ice. After they were anesthetized, their abdomen was cut open with a scalpel to collect hemolymph. A drop of hemolymph was placed onto a cell counting plate and counted microscopically. The remaining hemolymph was added with 200 μL of sterile PBS and mixed evenly. The mixture was centrifuged to obtain supernatant, and the latter was diluted gradiently at a ratio of 1:10. Then, 100 μL of each dilution was applied on a LB plate, incubated at 37°C for 24 h, and the colonies were counted. Bacterial load was calculated using the formula: bacterial load = number of colonies × dilution × 20 × 10 CFU/mL (37).

### Statistical analysis.

All experiments were independently conducted in triplicate. Statistical analysis was carried out using GraphPad Prism (version 6.02). Survival curves were plotted using the Kaplan-Meier method and comparison between groups was performed using the log-rank test. OD600 values were expressed as mean ± SD and statistical significance was assessed by One-way ANOVA. A *P*-value of less than 0.05 was considered statistically significant.

### Data availability.

The data that support the findings of this study are available on request from the corresponding author. The raw data and the assembled bacteriophage genome of BD523 have been uploaded to the BioProject database and can be downloaded at https://www.ncbi.nlm.nih.gov/bioproject/PRJNA881462 under accession number PRJNA881462.
