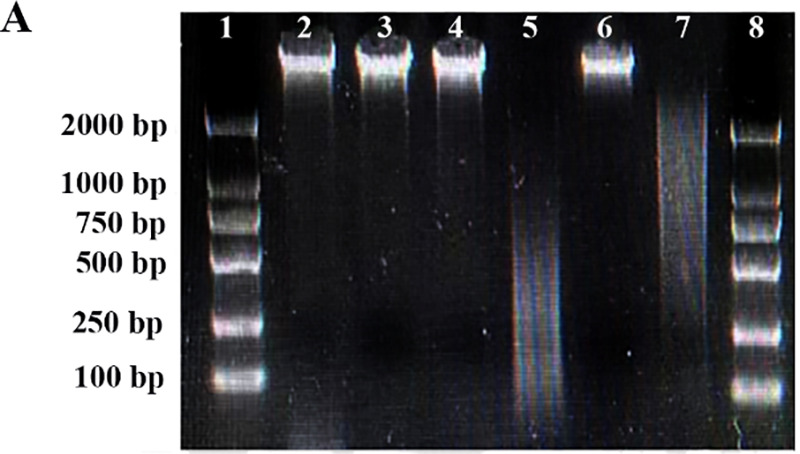# Erratum for Tao et al., “Synergistic Antibacterial Effect and Mechanism of Allicin and an Enterobacter cloacae Bacteriophage”

**DOI:** 10.1128/spectrum.02007-23

**Published:** 2023-06-26

**Authors:** Zhi Tao, Di Geng, Jiayue Tao, Jing Wang, Siqi Liu, Qiaoxia Wang, Feng Xu, Shengyuan Xiao, Rufeng Wang

## ERRATUM

Volume 11, no. 1, e03155-22, 2023, https://doi.org/10.1128/spectrum.03155-22. Page 3, Fig. 1: Due to incorrect labeling of electrophoresis lanes, panel A does not match its description in the figure legend. Panel A should appear as shown in this erratum. This error does not affect the original data, data interpretation, or conclusions of the article.[Fig fig1]

**Figure fig1:**