# Supplementary material for: Synergistic Antibacterial Effect and Mechanism of Allicin and an Enterobacter cloacae Bacteriophage
Source: Microbiol Spectr. 2022 Dec 6;11(1):e03155-22. doi: 10.1128/spectrum.03155-22 (PMC9927155; doi:10.1128/spectrum.03155-22)
Supplement: Supplemental file 1 — Fig. S1 to S10, Tables S1 to S6, and key resources table. Download spectrum.03155-22-s0001.pdf, PDF file, 1.6 MB [file spectrum.03155-22-s0001.pdf]

Supporting information for

Synergistic antibacterial effect and mechanism of allicin and an Enterobacter cloacae bacteriophage

Zhi Tao[1], Di Geng[1], Jiayue Tao[1], Jing Wang[1], Siqi Liu[1], Qiaoxia Wang[1], Feng Xu[2], Shengyuan Xiao[3], Rufeng Wang[1,*]

[1]School of Life Sciences, Beijing University of Chinese Medicine, Beijing 102488, China

[2]School of Pharmaceutical Sciences, Peking University Health Science Center, Beijing 100191, China

[3]Engineering Research Center of Edible and Medicinal Fungi, Ministry of Education, Jilin Agricultural University, Changchun 130118, China.

*Corresponding author: Rufeng Wang

Email: wrf@bucm.edu.cn (R.F.W.)

This supporting information includes the following:

1. Supplementary Figures S1 to S10
2. Supplementary Tables S1 to S6
3. Key resources table

1. Supplementary Figures

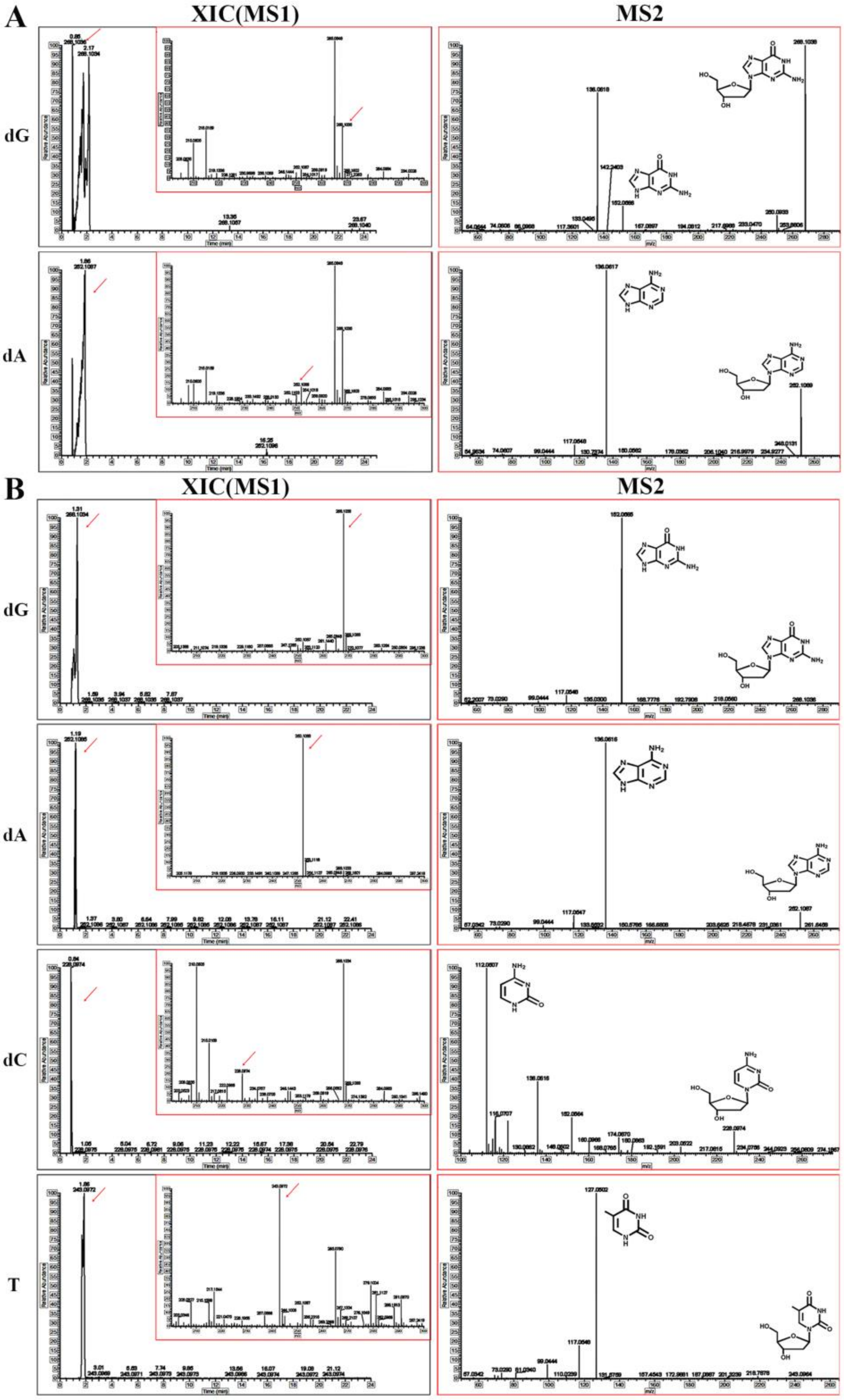

Fig. S1. Products of interaction between allicin and bacterial DNA or dNTPs detected by LC-MS/MS. (A) Parent ions and product ions for dG (*m/z* 268.1038 and 152) and dA (*m/z* 252.1089 and 136) are detected in reacting products of allicin with bacterial DNA. (B) Parent ions and daughter ions for dC (*m/z* 228.0974 and 112), dG (*m/z* 268.1036 and 152), dA (*m/z* 252.1087 and 136), and T (*m/z* 243.0964 and 127) are detected in reacting products of allicin with dNTPs. None of such ions is detected in the control without allicin added.

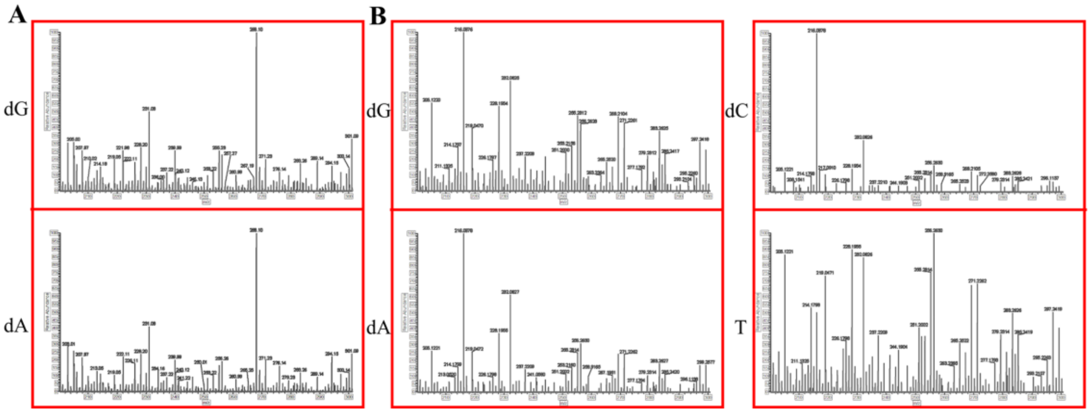

Fig. S2. Products of interaction between pure water (blank control) and bacterial DNA or dNTPs detected by LC-MS/MS. (A) Products of interaction between pure water and bacterial DNA. (B) Products of interaction between pure water and dNTPs. None of the parent and product ions of dC (*m/z* 228 and 112), dG (*m/z* 268 and 152), dA (*m/z* 252 and 136), and T (*m/z* 243 and 127) is detected.

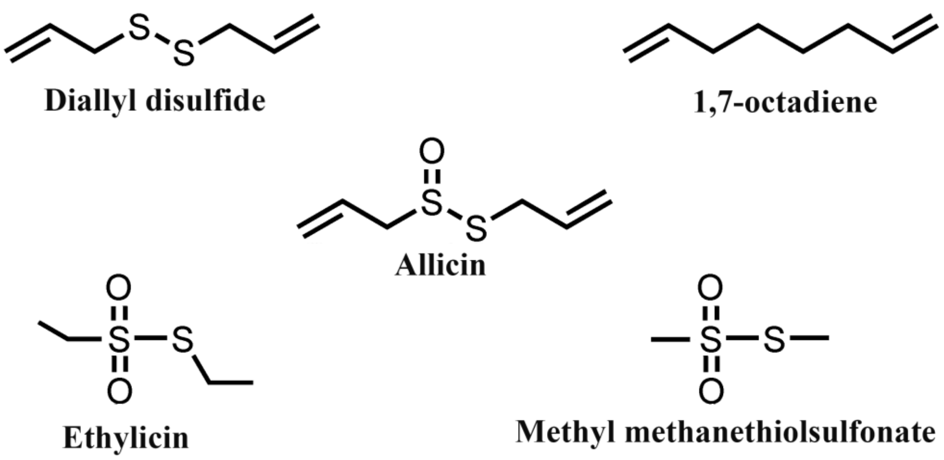

Fig. S3. Structures of allicin and its analogues.

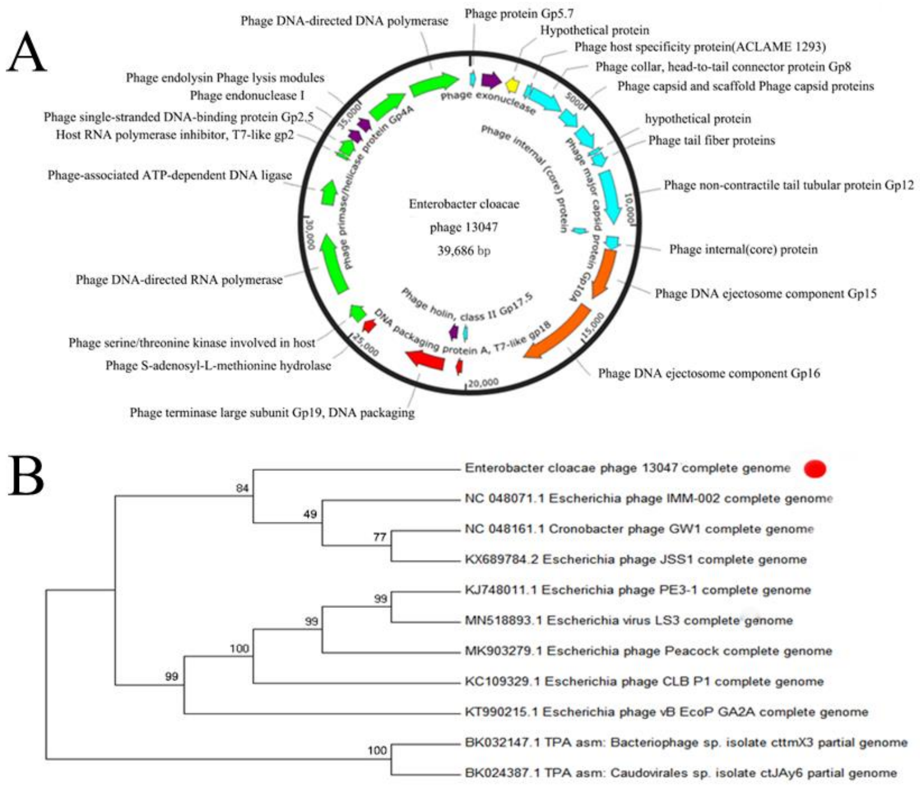

Fig. S4. Gene expression and cluster analysis of BD523. (A) Annotation of proteins expressed in genome of BD523. (B) Cluster analysis tree of BD523. The line with red spot designates BD523. Figures above branches represent bootstrap values.

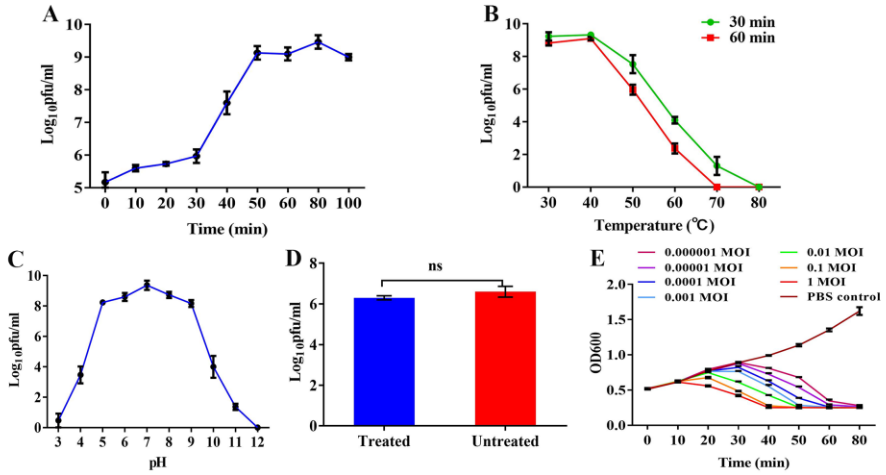

Fig. S5. Biological characteristics of BD523. (A) One-step growth curve of BD523. (B) Effect of temperature on BD523 titer at 30 and 60 min. (C) Effect of pH on BD523 titer. (D) Titers of BD523 before and after treated with chloroform for 30 min. (E) Bacteriostatic kinetics of BD523 at different MOI in 80 min. Values are expressed as mean ± SD (n = 3), statistical analysis was performed by One-way ANOVA and ns means statistically insignificant difference.

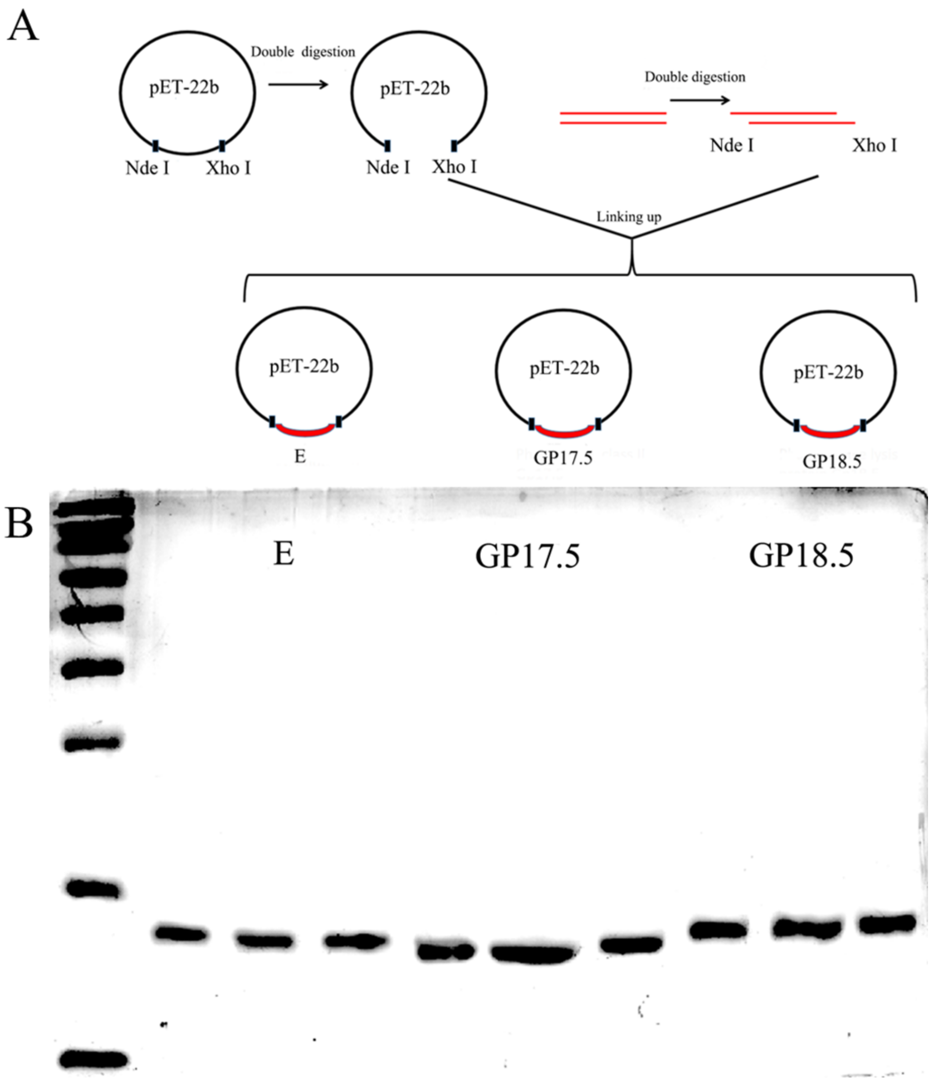

Fig. S6. Expression and detection of BD523 lyases. (A) Construction of recombinant plasmids for BD523 lyases. Plasmid pET22b and target genes (red) were double digested with Nde I and Xho I, and then linked up by ligase to obtain recombinant plasmids. (B) SDS-PAGE of BD523 lyases (n = 3). E designates the protein expressed by gene sequence E, and GP17.5 and GP18.5 designate the proteins expressed by gene sequences GP17.5 and GP18.5, respectively.

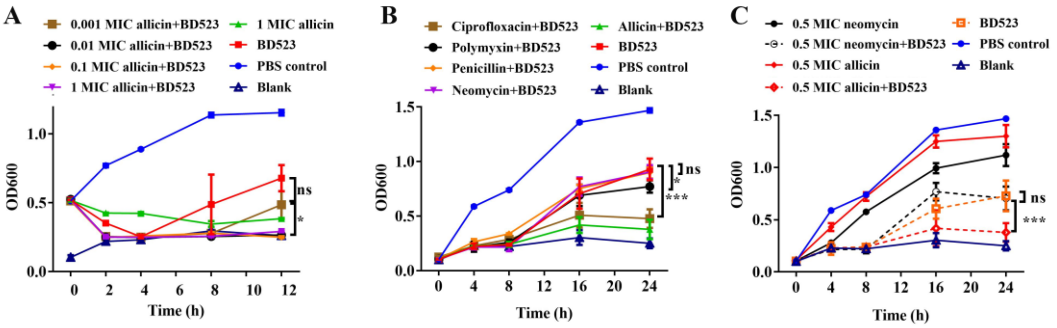

Fig. S7. Combined effects of BD523 and antibiotics against *E. cloacae*. (A) Combined effects of allicin at different MICs and BD523 in 12 h. (B) Antibacterial effect of antibiotics with different mechanism combined with BD523 in 24 h. (C) Combined effects of different antibiotics at 0.5 MIC and BD523 in 24 h. * means $p < 0.05$ and *** means $p < 0.001$ compared with the group connected by line, and ns means statistically insignificant difference.

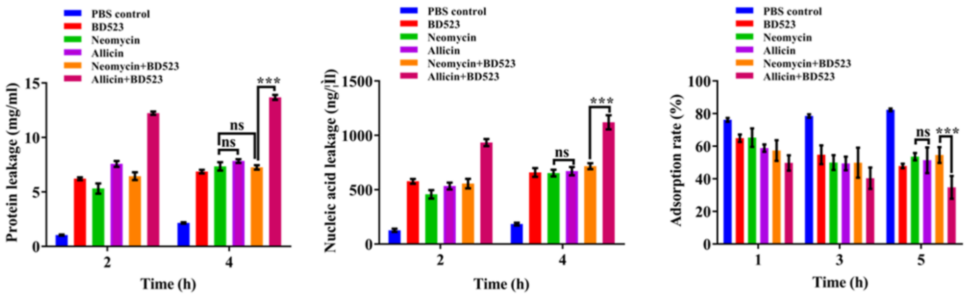

Fig. S8. Combined destructive effect of antibiotics and BD523 on cell wall and membrane of *E. cloacae*. (A) Proteins leakage in each group at 2 and 4 h. (B) Nucleic acids leakage in each group at 2 and 4 h. (C) Adsorption rate of bacterium determined with cetane on the basis of OD405 value in each group in 1, 3 and 5 h. All values are expressed as mean ± SD ($n = 3$) and statistical analysis was performed by One-way ANOVA. *** means $p < 0.001$ compared with the group connected by line, and ns means statistically insignificant difference. PBS means phosphate buffered saline.

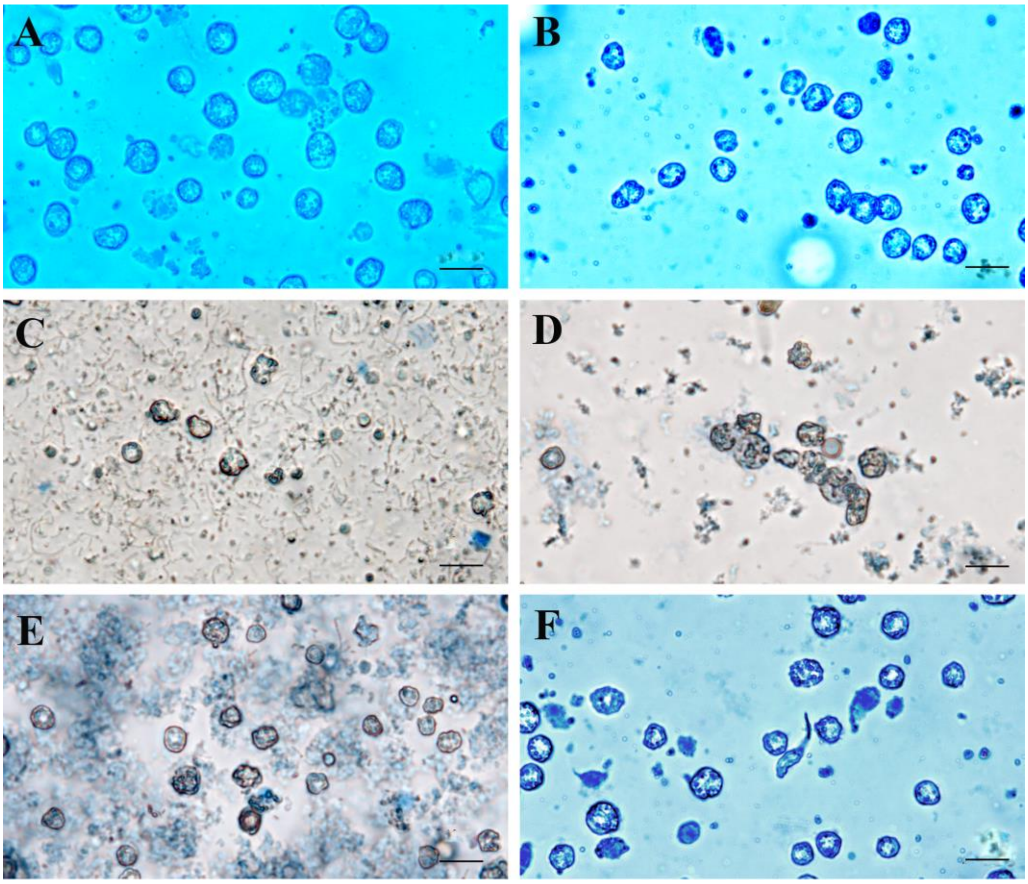

Fig. S9. Morphology of blood lymphocytes of *G. mellonella* in each group. (A) Blank control. (B) PBS control. (C) Model. (D) Treated with allicin. (E) Treated with BD523. (F) Treated with allicin + BD523. Bars are 10 μm long. PBS means phosphate buffered saline.

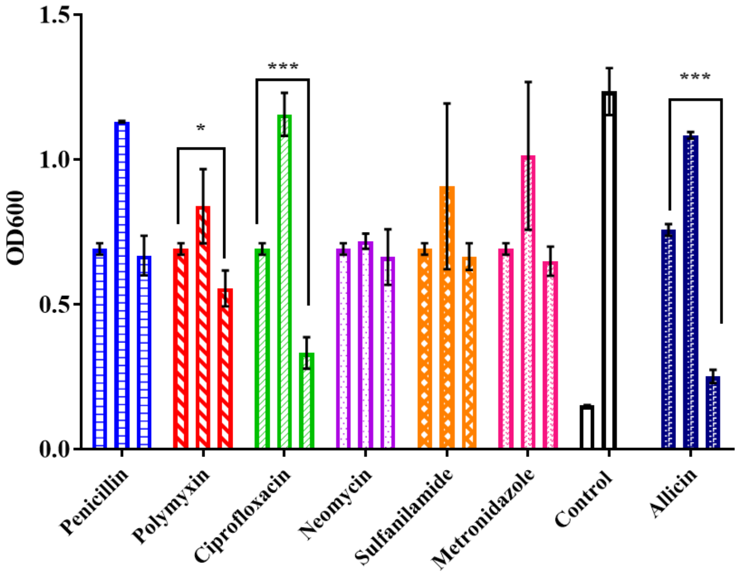

Fig. S10. Antibacterial effect of antibiotics combined with BD523 *in vitro*. The three bars of each color group from left to right are antibiotic group, model group, and antibiotic +BD523 group, respectively. Values are expressed as mean ± SD (n = 3), * means $p < 0.05$ and *** means $p < 0.001$ compared with the group connected by line.

2. Supplementary Tables

Table S1. Antibacterial activity of allicin, neomycin and vancomycin.

Antibiotics	Diameters of inhibition zone (mm)				
	**15.6 μg/ml**	**31.2 μg/ml**	**62.5 μg/ml**	**125 μg/ml**	**250 μg/ml**
Allicin	0	0	0	10.4±0.4	13.5±0.2
Neomycin	0	8.8±0.2	11.4±0.3	16.7±0.5	22.9±0.6
Vancomycin	0	0	0	0	10.1±0.3

Notes: Values are expressed as mean ± SD, $n = 3$.

Table S2. MIC of allicin and its structural analogues.

Compounds	OD600					
	**500 μg/ml**	**250 μg/ml**	**125 μg/ml**	**62.5 μg/ml**	**31.2 μg/ml**	**15.6 μg/ml**
Allicin	0.332±0.02	0.303±0.03	0.342±0.03 *	0.744±0.08	0.775±0.07	0.709±0.05
Ethylicin	0.261±0.01	0.201±0.03	0.26±0.08	0.291±0.07	0.239±0.05 *	0.582±0.12
1,7-octadiene	0.621±0.12	0.635±0.09	0.63±0.09	0.637±0.08	0.669±0.07	0.731±0.02
Methyl methanethiolsulfonate	0.213±0.07	0.232±0.01	0.194±0.05	0.173±0.02	0.261±0.04 *	0.543±0.05
Diallyl disulfide	0.345±0.08 *	0.693±0.09	0.539±0.12	0.535±0.14	0.661±0.10	0.614±0.07

Notes: Values are expressed as mean ± SD, $n = 3$. * means MIC.

Table S3. Amount, multiplicity of infection (OMOI) and titer of BD523 bacteriophage.

E. cloacae **(cfu/ml)**	**Amount (pfu/ml)**	**MOI**	**Titer (pfu/ml)**
1×10^{8}	1×10^{8}	1	0.802×10^{10}
1×10^{8}	1×10^{7}	0.1 *	0.894×10^{10}
1×10^{8}	1×10^{6}	0.01	0.398×10^{10}
1×10^{8}	1×10^{5}	0.001	0.186×10^{10}
1×10^{8}	1×10^{4}	0.0001	0.12×10^{10}
1×10^{8}	1×10^{3}	0.00001	0.196×10^{10}

Note: * means OMOI.

Table S4. Mass spectrometric parameters of compounds.

**Peak No.**	**Retention time (min)**	**Molecular formula**	**Parent ion (*m/z*)**	**Theoretical value (*m/z*)**	**Quality of error (ppm)**	**Product ion (*m/z*)**	**Appraisal result**
1	1.21	$C_{10}H_{13}N_5O_3$	252.1087	252.1091	-1.58	136.0616	Deoxyadenosine
2	1.88	$C_{10}H_{14}N_2O_5$	243.0964	243.0975	-4.52	127.0502	Thymidine
3	0.84	$C_9H_{13}N_3O_4$	228.0974	228.0979	-2.19	112.0507	Deoxycytidine
4	1.24	$C_{10}H_{13}N_5O_4$	268.1036	268.1040	-1.49	152.0565	Deoxyguanosine

Table S5. Target gene and primer sequence.

**Gene**	**Primer sequence**
Bacteriophage endolysin	F:AACTTTAAGAAGGAGATATACATATGGTGAGTAAGGTGCAATTCAAGCCGCGCAC<br>R:CTTGGTTGCGCTACAGTGGACGAAGATTGCGTCAGTGACTGTGCGCGGCTTGAAT
Bacteriophage holin, class II Gp17.5	F:TGTAGCGCAACCAAGCCGTCTCAGGACATCGGGGTAGACACTATCCGCATGTGGC<br>R:TGATGAAGTGGTAGCCAACGTCCAGCCAGCCCTGCTGTTTGTGCCACATGCGGATAG
Bacteriophage Rz-like lysis protein Gp18.5	F:GCTACCACTTCATCATCAAACGAGATGGTACTGTGGAAGAGGGACGCCCGGTCGATGTA<br>R:CCTACGGACCGGGAGTTCCAATCCTTAACGTGTGACCCTACTACATCGACCGGGCG

Table S6. Protein function of BD523 open reading frame

No.	Start	End	Length	Function
1	34	255	222	Bacteriophage protein Gp5.7
2	544	1410	867	Bacteriophage exonuclease
3	2125	1598	528	Hypothetical protein
4	2160	2528	369	Bacteriophage protein
5	2521	2769	249	Bacteriophage host specificity protein
6	2781	4349	1569	Bacteriophage collar, head-to-tail connector protein Gp8
7	4439	5323	885	Bacteriophage capsid and scaffold
8	5450	6499	1050	Bacteriophage major capsid protein Gp10A
9	6568	6762	195	Hypothetical protein
10	6823	7389	567	Bacteriophage tail fiber protein / T7-like tail tubular protein A
11	7389	7616	228	Bacteriophage protein
12	7628	9982	2355	Bacteriophage non-contractile tail tubular protein Gp12
13	10197	10054	144	Hypothetical protein
14	10240	10539	300	Bacteriophage internal (core) protein
15	10524	11108	585	Bacteriophage internal (core) protein

16	11120	13402	2283	Bacteriophage DNA ejectosome component, internal virion protein Gp15
17	13405	13566	162	Hypothetical protein
18	13848	17438	3591	Bacteriophage DNA ejectosome component Gp16, peptidoglycan lytic exotransglycosylase
19	17503	19956	2454	Hypothetical protein
20	19998	20207	210	Bacteriophage holin, class II Gp17.5
21	20189	20452	264	DNA packaging protein A, T7-like gp18
22	20557	21018	462	Bacteriophage Rz-like lysis protein Gp18.5
23	21003	22766	1764	Bacteriophage terminase large subunit Gp19, DNA packaging
24	23056	23214	159	Bacteriophage protein
25	24549	25001	453	Bacteriophage S-adenosyl-L-methionine hydrolase
26	25001	25177	177	Hypothetical protein
27	25199	25936	738	Bacteriophage serine/threonine kinase involved in host transcription shutoff Gp0.7
28	26104	26319	216	Bacteriophage protein
29	26319	26531	213	Hypothetical protein
30	26628	29294	2667	Bacteriophage DNA-directed RNA polymerase
31	29307	29540	234	Bacteriophage protein
32	29586	30050	465	Hypothetical protein

33	30093	30320	228	Bacteriophage protein
34	30324	30572	249	Bacteriophage protein
35	30572	31684	1113	Bacteriophage-associated ATP-dependent DNA ligase
36	31745	31864	120	Hypothetical protein
37	31914	32174	261	Bacteriophage protein
38	32167	32658	492	Hypothetical protein
39	32737	32901	165	Host RNA polymerase inhibitor, T7-like gp2
40	32945	33646	702	Bacteriophage single-stranded DNA-binding protein Gp2.5
41	33679	34116	438	Bacteriophage endonuclease I, four-way DNA junctions resolving
42	34095	34319	225	Bacteriophage protein
43	34309	34767	459	Bacteriophage endolysin
44	34782	34994	213	Bacteriophage protein
45	35023	36768	1746	Bacteriophage primase/helicase protein Gp4A
46	36817	36981	165	Bacteriophage protein
47	37053	39224	2172	Bacteriophage DNA-directed DNA polymerase
48	39224	39427	204	Hypothetical protein
49	39439	39684	246	Hypothetical protein

3. Key resources table

Reagent type (species) or resource	Designation	Source or reference
Strain, strain background	*Enterobacter cloacae*	Shanghai Beinuo Biotechnology Co., Ltd.
Chemical compound, drug	Allicin, diallyl disulfide	Shanghai Yuanye Bio-Technology Co., Ltd.
Chemical compound, drug	GelRed	Beijing Biorigin Biotechnology Co., Ltd.
Chemical compound, drug	Neomycin	Shanghai Aladdin Reagent Co., Ltd.
Chemical compound, drug	Vancomycin	Shanghai Macklin Biochemical Technology Co., Ltd.
Chemical compound, drug	1,7-octadiene, methyl methanethiolsulfonate	Shanghai Acmec Biochemical Technology Co., Ltd.
Chemical compound, drug	Luria Bertani medium	Beijing Mreda Technology Co., Ltd.
Chemical compound, drug	dNTPs	Sigma-Aldrich (Shanghai) Trading Co., Ltd.
Chemical compound, drug	PI	Wuhan AmyJet Scientific Technology Co., Ltd.
Chemical compound, drug	IPTG	Shanghai Thermo Fisher Scientific (China) Co., Ltd.
Commercial assay or kit	BCA Protein Assay Kit	Phygene Biological Technology Co., Ltd.
Commercial assay or kit	Bacterial DNA Extraction Kit	Beijing Biomed Gene Technology Co., Ltd.
Software, algorithm	Prism 6.02	GraphPad Software Co., Ltd.
Software, algorithm	RAST	www.rast.nmpdr.org
Software, algorithm	BLASTp	www.ncbi.nlm.nih.gov/BLAST/

Software, algorithm	SnapGene	Austin USA Insightful Science
Software, algorithm	MEGA 7.0	The National Institutes of Health
Software, algorithm	NanoAnalyze, ITC Run	Waters TA instruments Co., Ltd.
Software, algorithm	Chirascan	Shanghai Applied Photophysics Co., Ltd.
